# Seasonal and inter-annual variation of malaria parasite detection in wild chimpanzees

**DOI:** 10.1186/s12936-018-2187-7

**Published:** 2018-01-18

**Authors:** Doris F. Wu, Therese Löhrich, Andreas Sachse, Roger Mundry, Roman M. Wittig, Sébastien Calvignac-Spencer, Tobias Deschner, Fabian H. Leendertz

**Affiliations:** 1 Project Group Epidemiology of Highly Pathogenic Microorganisms, Robert Koch-Institut, Seestraße 10, 13353 Berlin, Germany; 2 Department of Primatology, Max Planck Institute for Evolutionary Anthropology, Deutscher Platz 6, 04103 Leipzig, Germany; 3 Max Planck Institute for Evolutionary Anthropology, Deutscher Platz 6, 04103 Leipzig, Germany; 4 Taï Chimpanzee Project, Centre Suisse de Recherches Scientifiques, BP 1303, Abidjan 01, Côte d’Ivoire

**Keywords:** *Plasmodium* spp., Malaria, *Pan troglodytes verus*, Temporal variation, Chimpanzees

## Abstract

**Background:**

Cross-sectional surveys of chimpanzee (*Pan troglodytes*) communities across sub-Saharan Africa show large geographical variation in malaria parasite (*Plasmodium* spp.) prevalence. The drivers leading to this apparent spatial heterogeneity may also be temporally dynamic but data on prevalence variation over time are missing for wild great apes. This study aims to fill this fundamental gap.

**Methods:**

Some 681 faecal samples were collected from 48 individuals of a group of habituated chimpanzees (Taï National Park, Côte d’Ivoire) across four non-consecutive sampling periods between 2005 and 2015.

**Results:**

Overall, 89 samples (13%) were PCR-positive for malaria parasite DNA. The proportion of positive samples ranged from 0 to 43% per month and 4 to 27% per sampling period. Generalized Linear Mixed Models detected significant seasonal and inter-annual variation, with seasonal increases during the wet seasons and apparently stochastic inter-annual variation. Younger individuals were also significantly more likely to test positive.

**Conclusions:**

These results highlight strong temporal fluctuations of malaria parasite detection rates in wild chimpanzees. They suggest that the identification of other drivers of malaria parasite prevalence will require longitudinal approaches and caution against purely cross-sectional studies, which may oversimplify the dynamics of this host-parasite system.

## Background

Six malaria parasite species (*Plasmodium* spp.) have been shown to infect wild chimpanzees (*Pan troglodytes*): *Plasmodium reichenowi*, *Plasmodium gaboni* and *Plasmodium billcollinsi* (subgenus *Laverania*), and the less common *Plasmodium vivax*-like*, Plasmodium ovale*-like and *Plasmodium malariae*-like parasites [1]. Another *Laverania* species of chimpanzee malaria parasites, *Plasmodium billbrayi*, is recognized by some, but not all, authors [1, 2]. The distribution, diversity and phylogenetic relationships of *Plasmodium* spp. have been the focus of several extensive studies conducted in the sub-Saharan African range of chimpanzees [1–5]. Although these studies did not aim at producing directly comparable prevalence estimates, the resulting picture was one of extreme geographic variations, with values ranging from 0% in *Pan troglodytes schweinfurthii* (Issa Valley, Tanzania) [5] up to 48% in *Pan troglodytes troglodytes* across multiple sites [6]. This spatial heterogeneity may be explained by a complex combination of ecological factors influencing malaria parasite transmission, including the availability and abundance of vectors and/or the demographic, social, and behavioural characteristics of chimpanzee communities [5, 7].

However, before the effect of such factors can be explored, clarifying temporal variation in *Plasmodium* spp. prevalence in chimpanzees is necessary. For humans, as well as birds (the key wildlife model in malaria research), longitudinal studies have shown that malaria parasite prevalence often varies significantly between seasons and years [8, 9]. Such unaccounted-for temporal variation may influence the apparent geographic distribution of chimpanzee malaria parasites (mainly deduced from cross-sectional sampling), complicating the identification of local drivers of transmission [5–7, 10]. This study aimed at filling a fundamental gap in the understanding of the basic epidemiology of malaria parasites in chimpanzees by determining whether *Plasmodium* spp. detection rate varied in a wild human-habituated community across four non-consecutive sampling periods.

## Methods

### Study site and sample collection

Faecal samples were collected from known individuals of one habituated chimpanzee (*Pan troglodytes verus*) community at Taï National Park, Côte d’Ivoire. The site experiences two annual wet (minor: September–October; major: March–June) and dry seasons (minor: July–August; major: November–February), with an annual average rainfall of 1800 mm and temperatures between 24 and 30 °C. In this study, 681 samples collected between 2005 and 2015 from 19 males and 29 females aged 1–52 years old were included. Samples were selected across four non-consecutive sampling periods (1: 2005–2006; 2: 2009; 3: 2011–2012; 4: 2014–2015). On average, 14.2 samples were selected per individual (range 3–37). As pregnancy correlates with increased *Plasmodium* spp. detection, known pregnant individuals were excluded [11]. Group size varied from 22 to 40. Samples were stored in liquid nitrogen within 12 h after collection, then shipped and stored in Germany at − 80 °C.

### Molecular analysis

DNA extracted from pea-sized faecal samples was tested for malaria parasites following a two-step screening process. First, samples were screened with a nested qPCR targeting a 90-bp fragment of a non-coding region of parasite mitochondrial DNA (mtDNA)—first round primers qPlasm1f 5′-CTGACTTCCTGGCTAAACTTCC-3′ and qPlasm1r 5′-CATGTGATCTAATTACAGAAYAGGA-3′ and second round primers qPlasm2f 5′-AGAAAACCGTCTATATTCATGTTTG-3′ and qPlasm2r 5′-ATAGACCGAACCTTGGACTC-3′ [11, 12]. Positive samples were then additionally screened using a semi-nested PCR targeting a longer 350-bp fragment of mtDNA that comprised the 3′ end of cytochrome oxidase 1 gene, a short intergenic region and the 5′ end of the cytochrome *b* gene—first round primers Plasmseq 1f 5′-GGATTTAATGTAATGCCTAGACGTA-3′ and Plasmseq 1r 5′-ATCTAAAACACCATCCACTCCAT-3′ and second round primers Pspcytbf1 5′-TGCCTAGACGTATTCCTGATTATCCAG-3′ and Plasmseq 1r [11, 12]. These PCR systems were previously shown to amplify DNA from a broad range of parasites belonging to the genera *Plasmodium* and *Hepatocystis* [11, 12]. As chimpanzees hunt, detection of prey infectious agents may result in false positives [13]. Therefore, samples without chimpanzee-specific parasite sequences (N = 24) were additionally tested for the chimpanzee’s main prey, colobines, targeting a 122-bp fragment of mitochondrial 12S ribosomal RNA gene (12S) [13]. If positive for colobines, samples (N = 5) were excluded from analyses as parasite origin was ambiguous. PCR products were sequenced on both strands via Sanger’s methodology and analysed using GENEIOUS v.10 [14]. Sequences were compared with publically available sequences using BLAST [15]. Samples were considered positive if: a) chimpanzee-specific parasite sequences were obtained from semi-nested PCR products (N = 70); or, b) samples were tested negative for colobines and a PCR product was obtained with the semi-nested PCR but the sequencing failed (N = 17) or sequence assignment was unspecific, e.g., *Plasmodium* sp. (N = 2) [11].

### Statistical analyses

To determine factors possibly affecting *Plasmodium* spp. detection in faeces, a Generalized Linear Mixed Model (GLMM) [16, 17] was fitted in R (R script available on request) [18] using R package lme4 (v.1.1.12, function glmer with a binomial error structure and logit link function [16, 19]). The full model included sex, age, (Julian) collection date, collection time, and sampling period as fixed effects. As the site experiences two wet and dry seasons per year, collection date (Julian date) expressed in radians was multiplied by two and the sine and cosine of it included (‘seasonality’) in the full model as additional fixed effects [20]. Individual was included as a random effect with age, collection date, collection time, and seasonality included as random slopes [21]. Age, collection date and collection time were z-transformed to mean of zero and standard deviation of one. To determine the combined significance of sex, age, sampling period, collection date, and seasonality, the full model was compared to a null model [22] lacking these fixed effects, but including all other terms using a likelihood ratio test (R-function anova with argument ‘test’ set as ‘Chisq’). Significance of each individual fixed effect was determined by comparing the full model with one lacking the respective fixed effect using a likelihood ratio test (R-function drop1 [22]). To test for seasonality, a reduced model without seasonality was compared to the full model using a likelihood ratio test. Total sample size for this analysis was 638 samples from 48 individuals. Model stability (assessed by dropping individuals one at a time and comparing model results with those obtained for the full model) [23] was not an issue.

## Results

### Molecular analysis

**Fig. 1:**
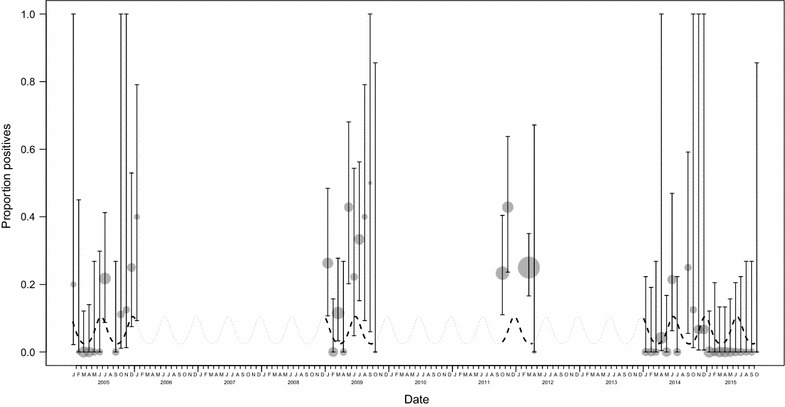
Temporal variation of malaria parasite faecal detection rate in chimpanzees. Circles represent monthly detection rates (with their 95% confidence intervals); circle area is proportional to the number of samples collected that month (range 1–85). The dotted line represents the model result regarding seasonal variation in detection rate

Overall, 89 samples (13%) were positive, with proportions of positive samples ranging from 0 to 43% per month and 4 to 27% per sampling period (Fig. 1). Sequences were obtained for 70 samples (79%). *Plasmodium* spp. sequences in this study exhibited 97–100% sequence identity to published sequences. The dominant *Plasmodium* spp. was *P. gaboni* (N = 47; 67.1%), followed by *P. billcollinsi* (N = 11; 15.7%), *P. reichenowi* (N = 6; 8.6%), *P. vivax*-like (N = 4; 5.7%), and *P. malariae*-like (N = 1; 1.4%). A single mixed infection (*P. gaboni* and *P. reichenowi;* 1.4%) was detected. During the study, 27 individuals (56%) were positive at least once (range: 1–9 positive samples per individual). In 16 of the 17 individuals who tested positive more than once different malaria parasite species were detected.

### Statistical analyses

The full model was clearly significant when compared to the null model (likelihood ratio test: *χ*^2^ = 65.997, df = 8, P < 0.001), specifically showing age and sampling period as significant predictors (Table 1). A decrease in detection probability was observed with increasing age (estimate + SE = − 0.691 + 0.249, *χ*^2^ = − 2.775, df = 1, P = 0.004). Seasonality (comparison of full and reduced model: *χ*^2^ = 9.240, df = 2, P = 0.010) was significant (Table 1) with peaks during June and December and lows during March and September (Fig. 1).

**Table 1:** Results of the GLMM

Term	Estimate	SE	*χ* ^2^	Df	P
(Intercept)	− 3.442	3.291			^a^
Sex	0.626	0.387	2.771	1	0.096
Age^e^	− 0.691	0.249	8.082	1	0.004
Collection date^e^	0.075	1.969	0.001	1	0.970
Sampling period 2^b^	1.531	2.149	43.657	3	< 0.001^c^
Sampling period 3^b^	1.862	3.645			
Sampling period 4^b^	− 0.928	5.163			
Collection date (sin)	− 0.353	0.227	9.240	2	0.010^d^
Collection date (cos)	0.707	0.290			
Collection time^e^	0.165	0.161	0.945	1	0.331

^a^Not indicated due to having a very limited interpretation

^b^Dummy coded with Sampling period 1 (2005–2006) as the reference category

^c^The indicated test refers to the overall effect of Sampling periods

^d^The indicated test refers to the overall effect of Seasonality

^e^z-transformed to a mean of zero and standard deviation of one; mean (sd) of original variables were 17.7 (13.1) years (age), 2011-05-26 (1307) days (collection date), and 10.9 (2.9) h (collection time), respectively

## Discussion

Marked, apparently stochastic variations in detection rates were observed at different temporal scales: monthly detection rates varied from 0 to 43%, whereas detection rates per sampling period varied from 4 to 27%. Strikingly, focusing on results of 2015 (N = 131) would have led to the erroneous conclusion that this chimpanzee community is malaria-free.

While shown for the first time in chimpanzees, such variations are not without precedent. Longitudinal studies of other host populations (e.g., humans, birds and lizards) showed both high seasonal and inter-annual variation, sometimes providing evidence for cyclical patterns with periods of 1–13 years [8, 9, 24–30]. In particular, large complex geographical variation in short and long-term patterns of *Plasmodium falciparum* has been observed worldwide [8]. For example, rates of malaria cases in Venezuela exhibited multi-annual cycles of 2–6 years, with higher rates directly following an El Niño event and increased rainfall [31]. Similarly, in Western Kenya, multi-annual malaria outbreak cycles of 2–4 years are associated with rainfall [24]. Such climatic factors are probably the strongest drivers behind prevalence patterns, having direct effects on vector abundance and parasite development and exhibiting high temporal fluidity [8, 9, 24, 28]. However, biotic factors most certainly also play a role, modifying the biology and behaviour of host, vector and/or parasite, and possibly operate at different temporal scales [7, 8, 24]. For example, changing host demographics with the influx of naïve and highly susceptible individuals can alter the pool of infective individuals, driving variability through changing processes of transmission and immunity within a population [24, 26, 31, 32]. The interplay between climatic and biotic factors therefore creates a spatiotemporally dynamic host-parasite system [8, 24].

Within a year, detection rates followed seasonal patterns: peaking 3 months after the start of the wet seasons and reaching their lowest levels at the end of dry seasons or beginning of wet seasons. Such seasonal increases in prevalence have been observed for most malaria parasites, including the closest relative of the chimpanzee-adapted Laveranian parasites, *P. falciparum* (the dominant cause of malaria-induced mortality in humans) [8]. In Bangladesh, both *P. falciparum* and *P. vivax* exhibited seasonal patterns associated with temperature and monthly rainfall [28]. This may be explained by higher abundance of vectors and/or higher prevalence of the parasites in vectors during rainy periods [8, 9, 24, 25]. The combination of stochastic and seasonal variation in detection rate, and presumably prevalence [8, 9, 27], has immediate practical consequences. These results caution against purely cross-sectional comparisons of detection rates across sites/habitat, which may over- or underestimate the abundance of parasites and oversimplify the dynamics of this host-parasite system.

Temporal variation also highlights the complexity of malaria parasite epidemiology in wild chimpanzee communities. This is partly due to the inherently complicated parasite life cycle and further complicated by fluctuating chimpanzee demography, community structure and behaviour [7]. For example, since younger individuals, and in particular infants and juveniles (under 4 and 10 years old, respectively), are more likely to be infected (this study and [5, 12]), changing group size and composition may alter infection patterns. As processes ultimately leading to the observed prevalence patterns are heterogeneous, future studies aimed at disentangling which of these factors determine the spatiotemporal distribution of malaria parasites in chimpanzees will require longitudinal sampling of a wide range of data on hosts, habitats, vectors, and parasite lineages [7–9, 27].

## Conclusions

This study shows strong temporal fluctuations of malaria parasite prevalence in wild chimpanzees. These findings call for longitudinal approaches to further characterize this host-parasite system and contribute to stress the resemblance with the human-*P. falciparum* system.
